# Graphene/Ferroelectric (Ge-Doped HfO_2_) Adaptable Transistors Acting as Reconfigurable Logic Gates

**DOI:** 10.3390/nano12020279

**Published:** 2022-01-17

**Authors:** Mircea Dragoman, Adrian Dinescu, Daniela Dragoman, Cătălin Palade, Valentin Şerban Teodorescu, Magdalena Lidia Ciurea

**Affiliations:** 1 National Institute for Research and Development in Microtechnology (IMT), Str. Erou Iancu Nicolae 126A, 077190 Voluntari, Romania; adrian.dinescu@imt.ro; 2 Faculty of Physics, Univ. of Bucharest, P.O. Box MG-11, 077125 Magurele, Romania; danieladragoman@yahoo.com; 3 Academy of Romanian Scientists, Str. Ilfov 3, 050094 Bucharest, Romania; teoval@infim.ro (V.Ş.T.); ciurea@infim.ro (M.L.C.); 4 National Institute of Materials Physics, Str. Atomistilor 405 A, 077125 Magurele, Romania; catalin.palade@infim.ro

**Keywords:** graphene, ferroelectrics, logical gates, intelligent transistors

## Abstract

We present an array of 225 field-effect transistors (FETs), where each of them has a graphene monolayer channel grown on a 3-layer deposited stack of 22 nm control HfO_2_/5 nm Ge-HfO_2_ intermediate layer/8 nm tunnel HfO_2_/*p*-Si substrate. The intermediate layer is ferroelectric and acts as a floating gate. All transistors have two top gates, while the *p*-Si substrate is acting as a back gate. We show that these FETs are acting memtransistors, working as two-input reconfigurable logic gates with memory, the type of the logic gate depending only on the values of the applied gate voltages and the choice of a threshold current.

## 1. Introduction

The memristor is a two-terminal nonlinear device with a hysteretic current–voltage dependence pinched at zero-voltage [1]. This hysteretic dependence rotates by sweeping the voltage a certain number of times, behavior that allows the memristor to memorize its previous state. Thus, a memristor is a non-volatile memory having an analogue behavior to a brain synapse since its conductance increases or decreases in time depending on the polarity of the applied voltage [2].

Memtransistors, on the other hand, are three-terminal memristor devices preserving all the above-mentioned physical properties of two-terminal memristors. They show a very low sneak-peak current when integrated in a crossbar array in comparison to two-terminal memristors [3], since the current in any memtransistor is controlled by one or several gates. A recent account about the physical principles and applications of memristors and memtransistors is found in [4].

The memtransistors hitherto reported are dominated by field-effect transistors (FETs) with MoS_2_ channels. The first reported MoS_2_ backgate memtransistor [5] shows memristive properties at a drain voltage of 80 V, which is too high for memory applications. This drain voltage value can decrease by up to 12 V by introducing top gate configurations [6] or using localized beam irradiation techniques [7]. Additionally, recent crossbar arrays of MoS_2_ memtransistors incorporating an array of 10 × 10 FETs [8,9] have been reported, paving the way for memtransistor applications in resistive memories and neuromorphic computation.

In all the above cases, the MoS_2_ channel has a thickness of 2–9 monolayers and a polycrystalline structure. The memristive effects are due solely to the modulation of the Schottky barrier at the drain and source terminals as a result of sulfur vacancy defects migration. Because the MoS_2_ memtransistors are based on the migration of sulfur defects and grain boundaries, their electrical properties are not reproducible from one device to another, since the distribution of such defects and boundaries are random and cannot be easily controlled. Therefore, we have adopted another solution for the conception and fabrication of memtransistors based on a ferroelectric FET, with a graphene monolayer channel [10,11] transferred onto a HfO_2_/Ge-HfO_2_/HfO_2_ ferroelectric structure grown on Si at the wafer scale [12]. This ferroelectric memtransistor works at very low drain voltages compared with the above devices, e.g., in the range of −2 V to +2 V. 

As mentioned above, the 3-layers-stack configuration below the graphene monolayer channel consists of control HfO_2_/Ge-HfO_2_ intermediate layer/tunnel HfO_2_/*p*-Si substrate, the intermediate layer having ferroelectric properties due to the combined effects of strain and doping induced by Ge quantum dots. Thus, due to the 5-nm-thick Ge-HfO_2_ layer, denoted further HfGeO thin film, we can have ferroelectricity and floating gate gathered in a single FET device, and can extend the fabrication of memtransistors at the wafer scale in a reproducible way. The floating gate is a classical and well-known solution for memory applications and transistor synapses [13], i.e., memtransistors.

As will be shown in the following, the three-gates configuration chosen for our graphene/HfGeO FETs, with two top gates and one backgate, allows for a single FET to act as several logic gates, depending on the voltages applied to the gates and the choice of current threshold. The study reported in this paper is thus at the forefront of the search for new transistor configurations, which guarantee that a single FET acts as a reconfigurable logic gate depending on the encoding of input and output states in various sets of gate voltages and, respectively, drain current values. Note that the implementation of OR and AND logic gates using single FETs containing a few monolayers of MoS_2_ flakes as channel and controlled by dual gates positioned as top- and backgates [14] (or two top gates [15]), has been demonstrated. In contrast, in the CMOS technology the basic logic gates are implemented using two FETs, and thus FETs having 2D material channels are able to reduce the footprint of transistors for implementation of digital logic gates. Additionally, if the gate dielectric is a ferroelectric, such as the polymer P(VDF-TrFE) [16], the memory and logic functions are taking place in the same device. All reconfigurable transistors based on 2D atomically thin materials flakes [17,18], or on CMOS technology [19], contain two or even three gates. We present below an array of 225 graphene/HfGeO FETs having two top gates and one backgate fabricated at wafer-scale. A single device functions as a reconfigurable logic gate.

## 2. Materials and Methods

The HfO_2_-based ferroelectric 3-layer thin film was prepared by magnetron sputtering deposition and consists of a stack of 22 nm control HfO_2_/5 nm Ge-HfO_2_ intermediate layer/8 nm tunnel HfO_2_/*p*-Si substrate (7–14 Ωcm). The growth procedure, the structural characterization of the 3-layer structure and PFM and DC measurements to evidence the ferroelectricity are reported in [12], and thus are not repeated here. 

**Figure 1:**
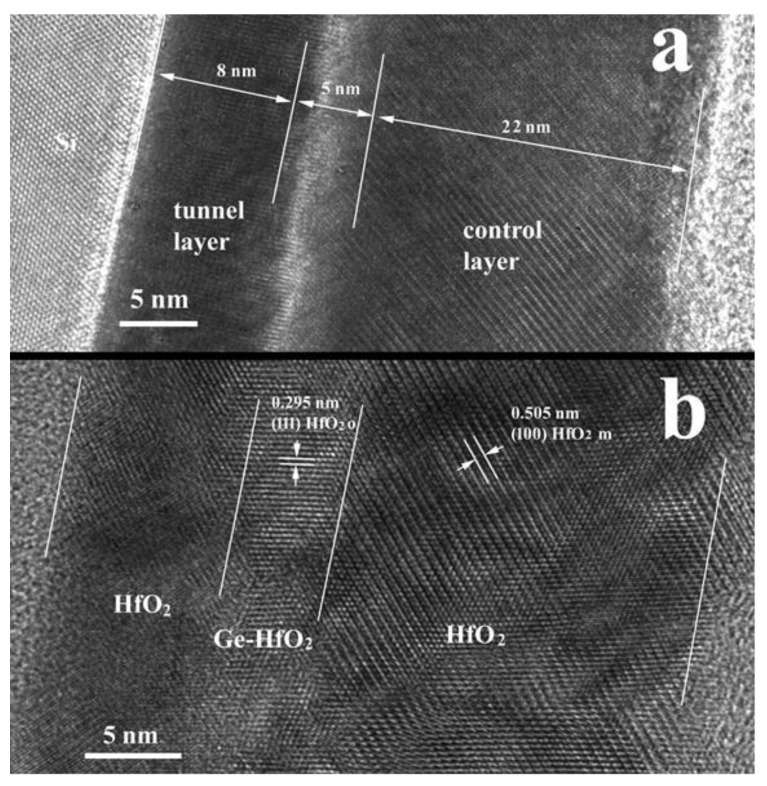
Cross section XTEM high resolution images of HfO_2_-based ferroelectric 3-layer structure; (**a**) image taken in the thick area of the XTEM specimen, revealing the region with high Ge concentration and (**b**) a similar image of a very thin area of the specimen revealing crystallization alignment of the HfO_2_ nanocrystallites in the 3-layer structure.

The HRTEM image of the thin film cross section, confirming the widths of the layers, is presented in Figure 1. The contrast in the image in Figure 1a is strongly correlated with the atomic Z number, and the white row in the Ge-HfO_2_ intermediate layer indicates the position of the high concentration of Ge atoms (including Ge dots). The contrast in the image in Figure 1b is mainly a phase contrast specific for HRTEM images. Here, we observe the presence of crystallographic correlation between the orthorhombic phase present in the mixed layer (HfO_2_o) and the monoclinic phase present in the control layer (HfO_2_m). Details of the origin of ferroelectric behaviour in the 3-layer stack can be found in [12].

Before device fabrication, the graphene monolayer was transferred by Graphenea, San Sebastian, Spain on a HfGeO/Si substrate with a dimension of 3 cm × 3 cm and Raman analysis was performed, telling us that about 85% of the wafer consists in graphene monolayers areas surrounded by grain boundaries where different types of graphene ranging from bilayers up to multilayers are found. 

**Figure 2:**
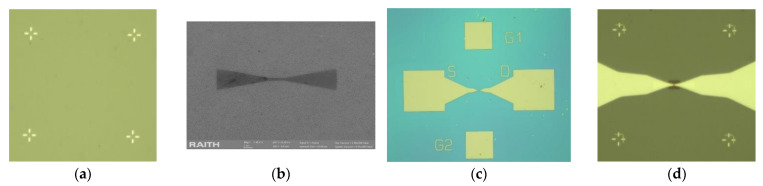
Optical images of the following fabrication steps: (**a**) alignment marks fabrication, (**b**) patterning using e-beam lithography, (**c**) source and drain contacts fabrication by e-beam, and (**d**) gate insulator deposition.

**Figure 3:**
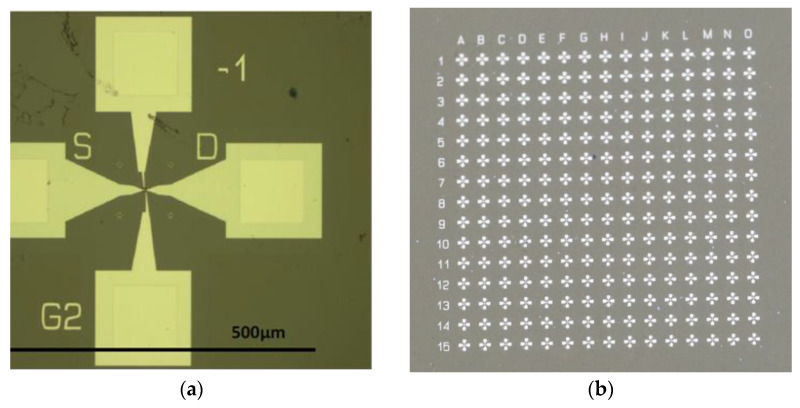
(**a**) Optical image of graphene/HfZrO FET with two top gates and a backgate (doped Si) and (**b**) the optical image of 225 graphene/HfZrO FET array.

The fabrication of the graphene monolayer/HfGeO devices at the wafer level is performed following these steps: (a) alignment marks fabrication, implying patterning (resist-PMMA 950k C2) using e-beam lithography (Raith e_Line, 30 k) followed by Ti (5 nm)/Au (95 nm) metal deposition by e-beam evaporation (Temescal FC 2000, Livermore, CA, USA) and lift-off, (b) graphene shaping by patterning the graphene channel using e-beam lithography (resist PMMA 950k A2) and RIE in O_2_ plasma, (c) source and drain contacts fabrication by e-beam lithography patterning (resist PMMA 950 k C2) and Ti (5 nm) /Au (35 nm) deposition by e-beam evaporation followed by lift-off, (d) gate insulator deposition patterning by e-beam lithography of the 40-nm-thick HSQ-(hydrogen silsesquioxane) gate insulator and the fabrication of metallic gate contacts. The optical images corresponding to the above-mentioned fabrication steps are displayed in Figure 2, while Figure 3a,b show the optical images of a single graphene/HfGeO FET with two top gates and a backgate (doped Si) and, respectively, the optical image of a 225 graphene/HfGeO FET array.

## 3. Results

The electrical characterization of graphene FETs was performed at room temperature using a Keithley SCS 4200 station, Keithley Instruments, Solon, OH, USA. The probe station for wafer measurement is placed inside a Faraday cage, and all electrical channels are connected with low-noise amplifiers to the station. The probe station is equipped with mechanical devices dedicated to attenuate mechanical vibrations and shocks. We have not used any fitting algorithms during or after the measurements, so the results depicted below are the measurements collected directly from the above equipment. 

**Figure 4:**
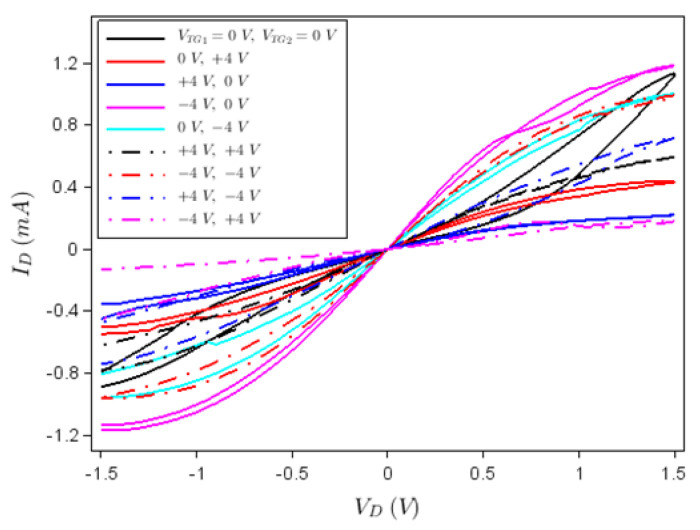
*I_D_*–*V_D_* dependence if *V_TG_*_1_ and *V_TG_*_2_ are connected while *V_BG_* is not connected.

**Figure 5:**
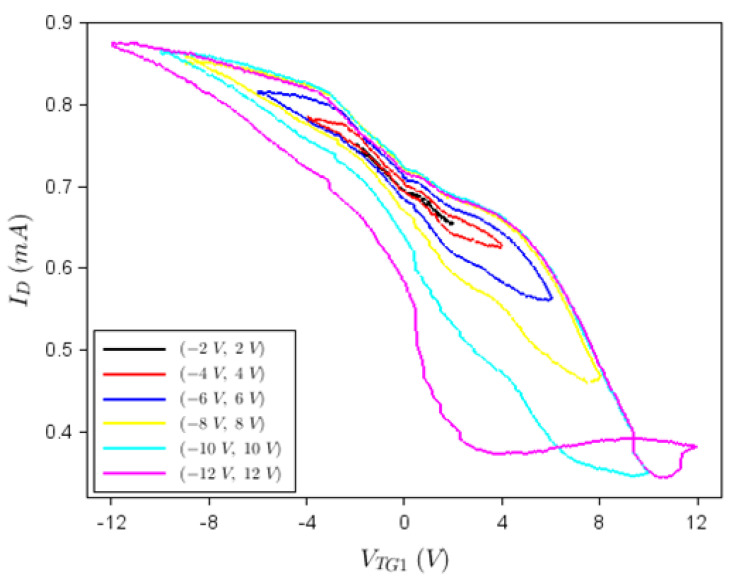
*I_D_*–*V_TG_*_1_ dependence while the other gates are not connected.

From the measured 225 transistors illustrated in the array in Figure 3b, 70% were found to work in a reproducible manner, the rest of them having various problems generated by bad metallic contacts, incomplete deposition and/or graphene defects; the measured errors of drain current versus drain voltage or gate voltage characteristics did not exceed 8%. A typical dependence of the drain current, *I_D_*, versus drain voltage, *V_D_*, at various top gate voltages, *V_TG_*_1_ and *V_TG_*_2_, is represented in Figure 4, while the back-gate voltage (*V_BG_*) is disconnected. In fact, Figure 4 shows that the device acts as a memtransistor due to the hysteretic clockwise behaviour of the *I_D_*–*V_D_* dependence at various top gate voltage values, the current in the forward drain voltage sweep (from 0 to 1.5 V) being higher than in the backward direction sweep (from 1.5 V to 0). This hysteretic behaviour is due to the ferroelectric HfGeO layer, which act as floating gate. A confirmation of this fact and of the memtransitor-like behaviour of our device is the *I_D_*–*V_TG_*_1_ dependence illustrated in Figure 5 and obtained by disconnecting the other gates; similar results were obtained by disconnecting any other two gates of the memtransistors. 

Figure 5, which shows that the transistor has a non-volatile memory conferred by the ferroelectric HfGeO layer, can be explained by the existence of polarized dipoles in the floating gate that control the concentration of charge carriers in the graphene channel. These charge carriers are holes because the graphene channel is initially *p*-doped due to technological processes related to the PMMA resist for e-beam channel patterning; as a consequence, the Fermi level is well below the Dirac point. As the voltage applied on the top gate changes from the minimum, negative value to 0, the concentration of holes (and hence the drain current) decreases, while the polarization intensity also decreases, the electric dipoles in the ferroelectric being oriented vertically, from the surface of the device toward the backgate. As *V_TG_*_1_ increases from 0 to positive voltages, the polarization gradually decreases to 0 and eventually switches sign (the polarization does not vanish when *V_TG_*_1_ = 0 due to the residual polarization in the ferroelectric), while the concentration of holes in graphene (and *I_D_*) reduces. The drain current has still positive values since the Fermi level does not cross the Dirac point. At inversing the sweep direction of *V_TG_*_1_ from positive to negative values, the flow of charge carriers in the graphene channel occurs in a similar way (including the existence of the residual polarization when *V_TG_*_1_ = 0), except that the electrical dipoles in the ferroelectric are oriented in the opposite direction; a detailed explanation of the hysteretic behavior in Figure 5 can be found in [20]. 

The graphene monolayer/ferroelectric FET is a thus a voltage-controlled transistor with a ferroelectric floating gate, i.e., the drain current is controlled by the gate voltages applied up and down the channel. In the case of 2D monolayer materials, as in our case, the drain current is only a surface current, which can be effectively controlled by the top- and backgate voltages. On the other hand, the ferroelectric floating gate confers memory properties to the same transistor. Thus, we find in a single device two functionalities: reconfigurability of digital logic gates, which can be implemented, and non-volatility of these logic gates until new gate voltages are applied.

The working of the memristor as reconfigurable logic gate can be understood with respect to the *I_D_–V_D_* characteristics in Figure 4. In this sense, the gates are programmed to have the values +4 V, −4 V and 0 V. The input for a logical gate level is considered as logic “0” if 0 V is applied, irrespective of the gate type, the logic value 1 being attributed otherwise. The output is encoded in *I_D_* (in the forward direction) for a drain voltage of 1 V; if we consider as reference/threshold the drain current level when *V_TG_*_1_ = *V_TG_*_2_ = 0 V, the drain current value above this threshold is associated to logic value 1, while the value below it is defined as logic value 0. Then, we have the logic table displayed as Table 1; the threshold drain current values for the OR and AND gates are 0.5 mA, whereas for XOR the corresponding value is 0.4 mA. The output could alternatively be considered the drain resistance *R_D_* which, at a certain voltage, increases and decreases in the same way as *I_D_*. Thus, we can attribute to *R_D_* the same logical value as to *I_D_* and, hence, we can implement in the same way the AND, OR and XOR logic gates. 

If we select the level (−4 V, +4 V) as 0 because it produces the lowest value of *I_D_* (below these gate voltages the transistor is OFF), we obtain a NAND gate, the functioning of which is detailed also in Table 1. For this logic gate, the threshold value for *I_D_* is 20 μA. NAND is a universal gate, in terms of which any logical gate or function can be expressed, and thus it is very important to implement this gate. All these results are obtained for two logical inputs (top gates) and one logical output–the drain current. Other logic gates can be implemented in a similar manner. 

Note that the reprogrammability of logic gates is entirely determined by the applied top- and/or backgate voltages, which impose the potential landscape throughout the ferroelectric layers. As such, whenever a gate voltage changes, the potential and hence the charge stored in the floating gate layer also changes. The role of the floating gate is to maintain the logic/charging state of the device between instants in which different signals are applied. 

**Figure 6:**
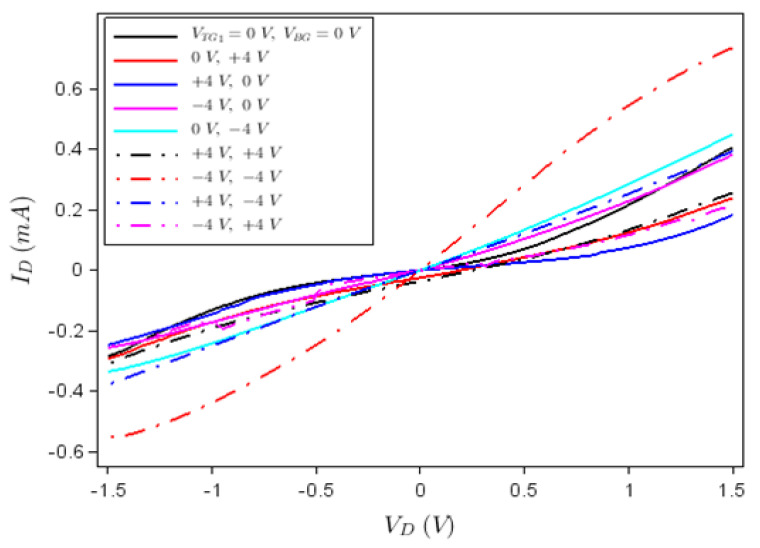
*I_D_*–*V_D_* dependence when *V_TG_*_2_ is not connected and *V_TG_*_1_ and *V_BG_* are connected.

Analogous results are obtained using a back gate and a top gate, for example *V_TG_*_1_ and *V_BG_*, while the other top gate is disconnected. The *I_D_–V_D_* dependence at various backgate voltages is shown in Figure 6 in the forward direction only, for simplicity. In this case, if we consider the drain current value at a certain drain voltage at *V_TG_*_1_ = *V_BG_* = 0 V as 0 logic, we can configure various logic gates as in the examples above. In principle, the 3-gate configuration of our FETs could allow for the implementation of reconfigurable logic gates having 3 inputs encoded in various gate voltage values. However, our experimental set-up has only four terminals, so that we could not simultaneously apply voltages on all 3 gates. The introduction of an additional terminal and voltage source would destroy the calibration of the apparatus.

**Figure 7:**
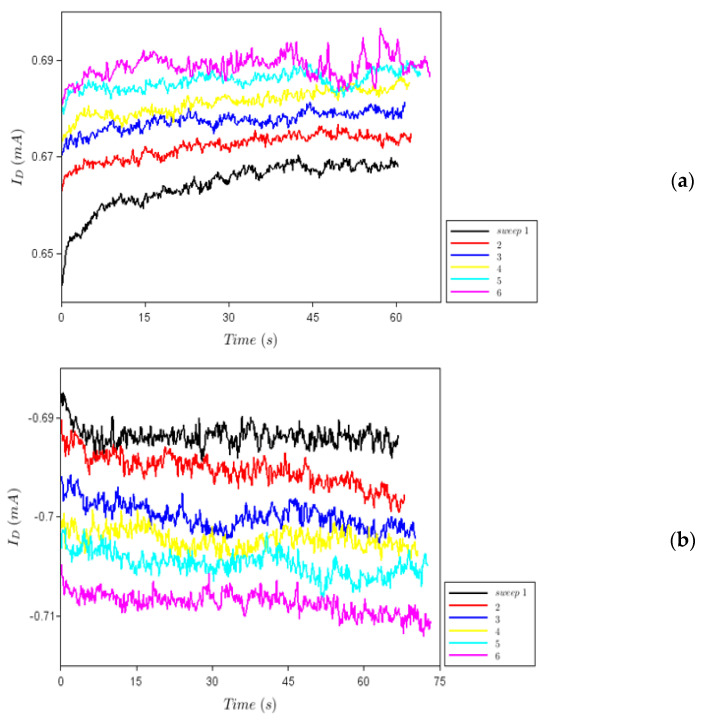
*I_D_*–time dependence at the drain voltages of (**a**) +1 V and (**b**) −1 V.

Figure 7a,b show the behaviour of *I_D_* in time for a positive (+1 V) and a negative (−1 V) drain voltage, respectively, at various voltage sweeps denoted by 1, 2, etc. We see that when a positive voltage is applied, the current increases and the conductance decreases, while the opposite occurs when the sign of the drain voltage is reversed. This is the typical behaviour of an electronic synapse, where the decreasing of conductance is attributed to potentiation, while the conductance increase is attributed to synapse depression [21,22].

A comparison of our results with similar results obtained for a dual-gate memtransistor having MoS_2_ as channel instead of graphene monolayer [8] is presented in Table 2. The parameters taken into account are the maximum drain current, *I_D_*_,max_, the maximum voltage values for the top and back gates, *V_TG_*_,max_ and *V_BG_*_,max_, the maximum drain voltage, *V_D_*_,max_, the on–off ratio, and the memory window. 

From Table 2, it follows that the memtransistors reported here and based on graphene have improved performances except for the on/off ratio. The on/off ratio in memtransistors with MoS_2_ channel is higher because MoS_2_ is a semiconductor with a bandgap of 1.3 eV, while suspended graphene is a zero-bandgap semiconductor, in which a bandgap of 0.3 eV is induced by the ferroelectric substrate (see Ref. [12] and the references therein). On the other hand, all other parameters reported in Table 2 are better compared to those in MoS_2_ memtransistors, since the growth and subsequent transfer of graphene on various substrates at the wafer scale is made industrially [23], while in the case of MoS_2_ these procedures are in infancy. Moreover, the fabrication procedures of graphene devices are more advanced compared to devices based on MoS_2_. Thus, a better control of growth, transfer and fabrication technologies reduces the number of defects in the graphene channel of memtransistors. 

## 4. Conclusions

We have shown that a three-gate field-effect transistor having as channel a graphene monolayer transferred over the ferroelectric structure HfO_2_/Ge-HfO_2_/HfO_2_ and fabricated at the wafer level is acting as a memtransistor. This single transistor is able to perform various basic 2-input digital logic gates, including universal gates. The type of the gate is programmed by the values of the gate voltages and of the threshold drain current. Recently, it was shown that a FET having a 2D semiconductor as channel and a top and a bottom gate is able to perform OR and AND operation when the channel was a few-layer MoS_2_ and XOR when the channel was WSe_2_ [24]. These FETs are termed as neuristors, since a single device is able to perform logical operations such as OR, AND and XOR, like a single cortex neuron [25]. However, the analogy with a neuron is incomplete since these neuristors have no memory, while in a neuron the memory and the various operations are performed in the same place [26]. 

In the devices reported in this manuscript, the memory and logical gates are located in the same device. However, we consider that the analogy between a transistor and a neuron is in its infancy, since the neuron has thousands of synapses connected to it and thus the advancement of solid-state intelligent matter [27] is at the beginning. Moreover, very recently, transistors such as those in this paper, able to perform different logical tasks as programmed, were referred to as adaptable or intelligent transistors [28]. Thus, our work could be an important step towards the development of artificial intelligence, which requires the fast adaptability of electron devices to various tasks.

## Figures and Tables

**Table 1:** Logic tables for different functions and their implementation. The inputs are written as (*V_TG_*_1_, *V_TG_*_2_ in [V])/logic values while the output is the logic value of *I_D_*.

OR		AND		XOR		NAND	
Inputs	Output	Inputs	Output	Inputs	Output	Inputs	Output
(0,0)/(0,0)	0	(0,0)/(0,0)	0	(0,0)/(0,0)	0	(−4,+4)/(0,0)	1
(0,−4)/(0,1)	1	(0,+4)/(0,1)	0	(0,−4)/(0,1)	1	(0,−4)/(0,1)	1
(−4,0)/(1,0)	1	(+4,0)/(1,0)	0	(−4,0)/(1,0)	1	(−4,0)/(1,0)	1
(−4,−4)/(1,1)	1	(−4,−4)/(1,1)	1	(−4,+4)/(1,1)	0	(−4,−4)/(1,1)	0

**Table 2:** Performance comparison between two memtransistors having as channel MoS_2_ and graphene.

Performances	Reference 8	This Work
*I_D_*_,max_ (mA)	0.1	0.8
*V_TG_*_,max_ (V)	±10	±12
*V_BG_*_,max_ (V)	±60	±4
*V_D_*_,max_ (V)	±40	±1.5
on/off ratio	10^5^	10^3^
memory window (V)	0.6	8

## Data Availability

Data presented in this paper could be made available upon request.
